# Interploidy hybridization in sympatric zones: the formation of *Epidendrum fulgens* × *E. puniceoluteum* hybrids (Epidendroideae, Orchidaceae)

**DOI:** 10.1002/ece3.752

**Published:** 2013-09-12

**Authors:** Ana P Moraes, Mariana Chinaglia, Clarisse Palma-Silva, Fábio Pinheiro

**Affiliations:** 1 Laboratório de Biossistemática e Evolução de Plantas, Departamento de Biologia Vegetal, Instituto de Biologia, Universidade Estadual de Campinas/UNICAMPCampinas, São Paulo, Brasil; 2 Programa de Pós Graduação em Evolução e Diversidade, Universidade Federal do ABC/UFABCSanto André, São Paulo, Brasil; 3 Departamento de Ecologia, Instituto de Biologia, Universidade Estadual Paulista/UNESPRio Claro, São Paulo, Brasil; 4 Instituto de Botânica, Núcleo de Pesquisas do Orquidário do EstadoSão Paulo, Brasil

**Keywords:** *Epidendrum*, GISH, hybrid zone, interploidy crossing, karyotype, orchids, plant speciation

## Abstract

Interspecific hybridization is a primary cause of extensive morphological and chromosomal variation and plays an important role in plant species diversification. However, the role of interploidal hybridization in the formation of hybrid swarms is less clear. *Epidendrum* encompasses wide variation in chromosome number and lacks strong premating barriers, making the genus a good model for clarifying the role of chromosomes in postzygotic barriers in interploidal hybrids. In this sense, hybrids from the interploidal sympatric zone between *E. fulgens* (2*n* = 2*x* = 24) and *E. puniceoluteum* (2*n* = 4*x* = 56) were analyzed using cytogenetic techniques to elucidate the formation and establishment of interploidal hybrids. Hybrids were not a uniform group: two chromosome numbers were observed, with the variation being a consequence of severe hybrid meiotic abnormalities and backcrossing with *E. puniceoluteum*. The hybrids were triploids (2*n* = 3*x* = 38 and 40) and despite the occurrence of enormous meiotic problems associated with triploidy, the hybrids were able to backcross, producing successful hybrid individuals with broad ecological distributions. In spite of the nonpolyploidization of the hybrid, its formation is a long-term evolutionary process rather than a product of a recent disturbance, and considering other sympatric zones in *Epidendrum*, these events could be recurrent.

## Introduction

Interspecific hybridization is frequently cited as a primary cause of the extensive morphological and chromosomal variation observed in plant genera and species complexes (Petit et al. 1999; Chapman and Abbott 2010; Souza et al. 2012; Presgraves 2013), especially in sympatric areas. The merging of two divergent genomes into a unique nucleus, caused by hybridization, can set the stage for dynamic changes in the genome, transcriptome, and phenotype of the new hybrids, what could have consequences on parental species after hybrid backcrossing species (Leitch and Leitch 2008; Soltis et al. 2009; Paun et al. 2010, 2011a; Jiao et al. 2011; Buggs et al. 2012). This event is also responsible for increasing the genetic diversity within species, transferring genetic adaptations between species, breaking down or reinforcing reproductive barriers between closely related groups, which can lead to the emergence of new ecotypes or species, and playing a role in the adaptive radiation of plant species (Soltis et al. 2003; Slotte et al. 2008; Jorgensen et al. 2011).

Hybridization events have been repeatedly observed among food-deceptive orchids as *Epidendrum* L., the largest Orchidaceae genus in the Neotropical region, representing *c*. 1500 species distributed from Florida (USA) to Argentina (Hágsater and Soto-Arenas 2005; Pinheiro and Cozzolino 2013). Generally, *Epidendrum* species lack strong premating barriers because they have high interspecific reproductive compatibility, share extensive numbers of pollinator species, and frequently have overlapping flowering periods (Almeida and Figueiredo 2003; Hágsater and Soto-Arenas 2005; Pansarin and Amaral 2008). These characteristics favor the formation of hybrid swarms, such as those commonly observed in sympatric species from the subgenus *Amphyglottium* (Dunsterville 1979; Dressler 1989; Hágsater and Soto-Arenas 2005; Pinheiro et al. 2010). Taxonomic problems within this group are probably the result of hybridization and late generation introgression among co-occurring taxa, which blur species boundaries and increase morphological variability (Pinheiro et al. 2010).

Hybridization may also be associated with the origin of the extensive chromosomal variation observed within *Epidendrum*, ranging from 2*n* = 24 (*E. fulgens* Brongn.) to 2*n* = 240 (*E. cinnabarinum* Salzm. ex Lindl.), with dysploid variation between extremes (Tanaka and Kamemoto 1984; Pinheiro et al. 2009; Felix and Guerra 2010). Changes in chromosome number (Rieseberg 2001; Cozzolino et al. 2004; Rieseberg and Willis 2007) and genetic incompatibilities (Lexer and Widmer 2008; Scopece et al. 2010) may act as important postzygotic barriers in food-deceptive orchids, which are pollinated by a broad assemblage of flower visitors (Moccia et al. 2007; Scopece et al. 2008).

**Figure 1:**
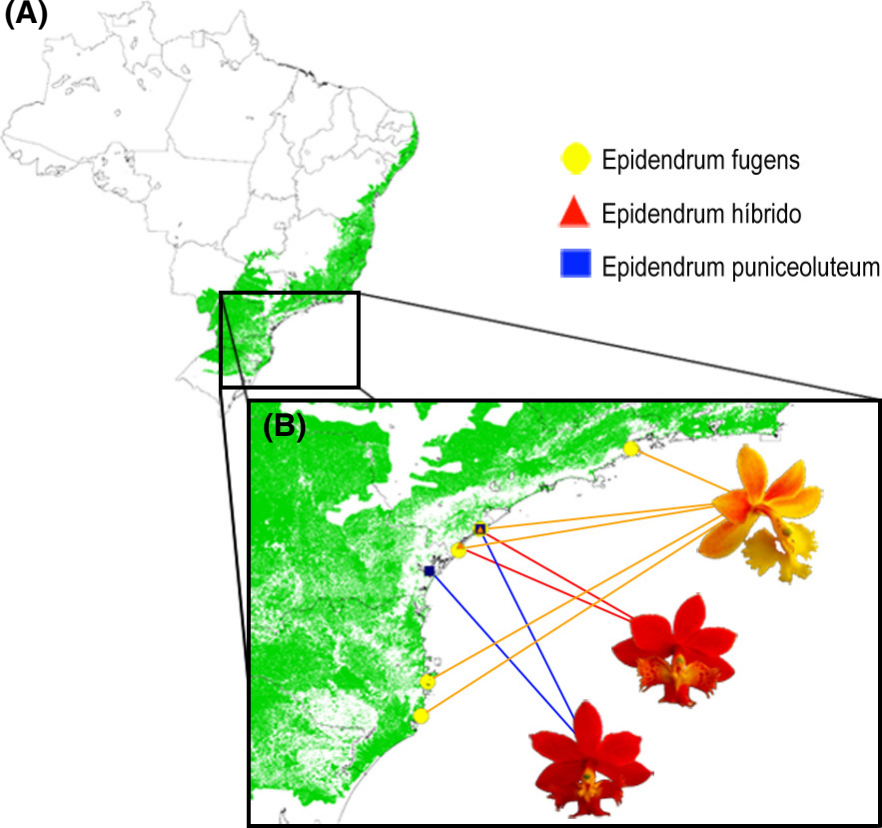
*Epidendrum fulgens* and *E. puniceoluteum* sympatric zone. (A) Map of Brazil with the Atlantic Rain Forest marked in green. (B) Detail from the sympatric zone with collection points indicated by flowers. Typical flower colors for the species and color variations in hybrid individuals from the sympatric zone are indicated. Map adapted from Pinheiro et al. (2010) and morphological data from Pinheiro and Barros (2006).

*Epidendrum* species present various sympatric zones throughout the distribution of the genus, and the one composed by *E. fulgens* (2*n* = 2*x* = 24) and *E. puniceoluteum* F. Pinheiro & F. Barros (2*n* = 4*x* = 52) should be highlighted (Tanaka and Kamemoto 1984; Pinheiro et al. 2009). This sympatric zone extends from the south to south-eastern coastal plains in Brazil (Fig. 1), with *E. fulgens* colonizing sand dunes and *E. puniceoluteum* swampy areas. *Epidendrum fulgens* and *E. puniceoluteum* have distinct flower color combinations (orange sepals and petals with yellow with red dots labellum in *E. fulgens*; red sepals, petals, and labellum with a yellow–orange callus in *E. puniceoluteum*), but throughout the sympatric zone, gradual color variation can be observed from one extreme to the other (Pinheiro and Barros 2006; Fig. 1), and these intermediary individuals can be observed throughout sand dunes and swampy areas (Pinheiro et al. 2010). Different butterfly and moth species are known to pollinate *Epidendrum* species (Pansarin and Amaral 2008), and on food-deceit systems as observed on *Epidendrum*, such variable flower color could be bennefical, to puzzel the pollinator. Previous analyses of nuclear and plastid markers and manual crossings have confirmed F1 and F2 hybrid formation and it backcrossing toward the polyploid specie *E. puniceoluteum*, but not toward the diploid *E. fulgens* (Pinheiro et al. 2010).

The difference in chromosome number and ploidy level between *E. fulgens* and *E. puniceoluteum* makes this sympatric zone even more challenging because hybrid fertility decreases as the difference in parental chromosome number increases (Levin 2002). The importance of interploidal hybridization for generating viable, fertile wild hybrids, and increasing population diversity and gene introgression among parental species is well documented (Stebbins 1971; Petit et al. 1999; Flatberg et al. 2006; Slotte et al. 2008; Chapman and Abbott 2010; Ricca et al. 2011). Examples in Orchidaceae are well reported in the literature, especially in the genus *Dactiylohriza* Neck. ex Nevski on crossings involving *D. fuchsii* (2*n* = 40) with *D. purpurella* and *D. praetermissa* (both 2*n* = 80; cited as *Dactylorchis* in Heslop-Harrison 1953, 1957); *D. fuchsii* × *D. maculata* (2*n* = 80) (Ståhlberg 2009), *D. incarnata* (2*n* = 40) × *D. praetermissa* (De Hert et al. 2011) and even triple hybrids among *D. fuchsii* (2*n* = 40), *D. incarnata* and *D. praetermissa*) (De hert et al. 2012).

The behavior of chromosomes in a new hybrid nucleus can be analyzed through chromosome characterization techniques as chromosome banding and *in situ* hybridization direct localizing DNA sequences on chromosomes (Jiang and Gill 2006; Chester et al. 2010). The complete genome is useful for differentiating parental chromosomes in a cell of a hybrid individual through genomic *in situ* hybridization (GISH) techniques (Schwarzacher et al. 1989; Markova et al. 2007; Markova and Vyskot 2009). The success of GISH in demonstrating the two parental genomes depends on two main factors. First, the amount of genome differentiation that exists between the parents – more related genomes are harder to differentiate in the hybrid chromosomes. Second, the age of the hybrid – older hybrids suffer more complete genome turnover, diverging from the parental sequences. The divergence can be so profound that the hybrid genome does not match either parent. However, the time required for genome complete restructuring is thought to be long, for example, *c*. 5 million years in *Nicotiana* (Leitch et al. 2008; Koukalova et al. 2010).

In this sense, sympatric zones and natural hybrids formed between *E. fulgens* and *E. puniceoluteum* represent an excellent model for investigating the evolutionary significance of chromosome rearrangements and the role of postzygotic barriers, such as meiosis normality, pollen fertility, and pollen tube growth. To accomplish our objective we applied (1) meiotic analysis on both parental and hybrid individuals to verify meiotic normality, (2) pollinium analysis to estimate pollen viability and pollen tube grown, (3) karyotype analysis – chromosome number, chromosome banding, and *in situ* hybridization – to evaluate karyotype constancy throughout the sympatric zones and karyotype parental × hybrid relatedness and GISH analysis to determine the contribution of each parental to hybrid formation and possible causes of genome variation among hybrids.

## Materials and Methods

### Plant material

Wild specimens of *E. fulgens*, *E. puniceoluteum,* and their hybrids were collected from sympatric populations found throughout south and south-eastern Brazil (Table 1). The same individuals were previously genotyped by Pinheiro et al. (2010); nuclear and plastid microsatellites were used to detect pure parental genotypes and hybrid individuals. Collection points were georeferenced and plotted on a map using DivaGis 7.5 (http://www.diga-gis.org.br; Fig. 1). The collected individuals were cultivated in the living orchid collection at the Instituto de Botânica, São Paulo, Brazil. Vouchers were deposited at the herbarium SP.

**Table 1:** *Epidendrum* microsporogenesis – meiosis normality and pollinium viability

Species^1^	Population	No. plants	*n*	Meiosis		Pollinarium viable (%)	Viable cells (%)

				No. cells	Regular cells (%)	Slides (no. cells)	
*E. fulgens*	Paraty/RJ	2	12	1000	99.55	–	–
	Cananéia/SP	2		1000	98.90	8 (4000)	99.78^a^
	Ilha Cardoso/SP	1		500	98.40	–	–
	Imbituba/SC	1		500	99.60	–	–
	Total			3000	99.15	–	–
*E. puniceoluteum*	Cananéia/SP	2	28	1000	99.15	–	–
	Paranaguá/PR	2		1000	98.60	–	–
	Ilha Comprida/SP	4		2000	97.085	8 (4000)	99.24^a,b^
	Total			4000	97.74	–	–
Hybrid	Cananéia/SP	3	19^2^	1500	29.6	–	–
	Ilha Comprida/SP	10		6000	8.68	15 (7500)	98.99^b^
	Total			7500	12.85		

Viable cells means followed by the same letter are not significantly different (Student*–*Newman–Keuls test, *P* < 0.05).

1 Species were identified by SSRs following Pinheiro et al. (2010).

2 The chromosome number could only be defined on one slide.

### Meiosis analysis, pollen viability, and pollen tube germination

To obtain pollen mother cells (PMCs) that were undergoing meiosis, young floral buds were fixed in ethanol:acetic acid (3:1, v/v) for 24 h at room temperature and stored at –20°C. To evaluate the meiotic process, pollinia were washed two times in distilled water, digested in 2% (w/v) cellulase (Serva, Heidelberg, Germany)/20% (v/v) pectinase (Sigma, St. Louis, MO) at 37°C for 5 min and squashed in a drop of 60% acetic acid. The best slides were frozen in liquid nitrogen to aid coverslip removal, stained using a solution of 2% (w/v) Giemsa (Sigma) for 5 min, and mounted in a drop of Entellan® (Merk, Darmstadt, Germany). The slides were analyzed to evaluate the meiotic normality by the means of frequency of cells without meiotic abnormalities, for example, pairing errors, segregation errors, and presence of micronuclei or tetrads with less/more four cells.

Tetrad stainability and morphology were used to estimate pollen viability, following Alexander (1980). Three flowers from eight pure individuals of *E. fulgens* and *E. puniceoluteum* and from 15 hybrid individuals from the Ilha Comprida population, all classified by simple sequence repeats (SSRs) following Pinheiro et al. (2010), were collected at anthesis and their pollinia were removed and fixed in ethanol:acetic acid (3:1, v/v) for 24 h at room temperature and stored at −20°C. Three slides were prepared from each individual and the first 500 tetrads were classified. The average frequency of meiotic normality (cells carrying out a normal meiosis) and pollen viability among the parental species and hybrids were analyzed using a nonparametric Kruskal–Wallis test followed by a Student*–*Newman–Keuls test using BioEstat 5.0 (Ayres et al. 2007).

### Pollen tube analyses

Hand-pollination experiments were conducted to assess the strength of postpollination barriers. To evaluate the compatibility in crossings involving hybrids and parental individuals, controlled pollination experiments were conducted in the orchid nursery of Frederico Carlos Hoehne at the Instituto de Botânica de São Paulo, São Paulo, Brazil. Flower buds in preanthesis were isolated with paper bags to exclude floral visitors. For these experiments, five flowers per plant (four *E. fulgens*, six *E. puniceoluteum,* and eight hybrid specimens) were used for each pollination treatment. The following pollination experiments were performed: (1) positive control – flowers from each parental species were cross-pollinated with pollinaria from another individual from the same species, with two crossings for each parent; (2) hybrid cross-pollination – hybrid flowers were pollinated using pollinaria from other hybrid plants in three different crossings; (3) hybrid × parental cross-pollinations – *E. puniceoluteum* and *E. fulgens* flowers were pollinated using hybrid pollinaria, with two individuals/parental species for each crossing; and (4) parental-hybrid cross-pollination – hybrid flowers were pollinated using *E. fulgens* and *E. puniceoluteum* pollinaria, with three different individuals/parental species for each crossing. Flowers were collected at 3, 5, 7, 9, and 12 days after pollination and stored in 70% ethanol. Pistils were stained with aniline blue to aid the observation of pollen tube growth under a fluorescence microscope (Martin 1959), using a WU filter from Olympus (Tokyo, Japan).

### Mitosis analysis

#### Pretreatment and storage

Root tips were pretreated in 8-hydroxyquinoline (0.002 mol/L) for 24 h at 10°C, fixed in ethanol:acetic acid (3:1, v/v) for 24 h at room temperature and stored at −20°C.

#### Chromosome counting and karyotyping

Chromosome preparations were performed by conventional staining following Guerra (1983). Fixed root tips were washed in distilled water three times for 5 min each, hydrolyzed in 5 N HCl at room temperature for 20 min and transferred to distilled water until they were squashed in a drop of 45% acetic acid. The best slides were frozen in liquid nitrogen and the coverslip was removed. The selected slides were stained using 2% (w/v) Giemsa (Sigma) for 5 min and mounted in a drop of Entellan® (Merk).

#### Chromosome banding

Fixed root tips were washed in distilled water and digested in a 2% (w/v) cellulase (Serva)/20% (v/v) pectinase (Sigma)/1% macerozyme (Sigma) solution at 37°C for 30 min. The meristem was squashed in a drop of 45% acetic acid and the coverslip was later removed in liquid nitrogen. After 3 days, the preparations were stained with chromomycin A_3_ (CMA; 0.5 mg mL^−1^) for 1 h and counterstained with 4′,6-diamidino-2-phenylindole (DAPI; 1 μg mL^−1^) for 30 min. After the analysis, the best slides were destained and stored for fluorescence in situ hybridization (FISH) and GISH.

#### DNA probes, FISH, and GISH

A D2 probe from *Lotus japonicus* (Regel) K. Larsen (Pedrosa et al. 2002) and an R2 probe from *Arabidopsis thaliana* (L.) Heynh. (Wanzebock et al. 1997) were used to localize 5S and 45S rDNA, respectively. Briefly, the 5S rDNA probe was labeled with digoxigenin-11-dUTP and the 45S rDNA probe was labeled with biotin-14-dUPT, both by nick-translation (Roche Biochemicals, Burgess Hill, West Sussex, UK). In situ hybridization was performed at 77% stringency using a mixture of 50% (v/v) formamide, 10% (w/v) dextran sulfate, and 0.1% (w/v) sodium dodecyl sulfate in 2× saline-sodium citrate buffer (SSC) with 3–5 ng μL^−1^ of each probe. After overnight hybridization at 37°C, the slides were washed in 2× SSC and 0.1× SSC (two washes). The 5S rDNA probe was detected with antidigoxigenin conjugated to rhodamine (Roche Biochemicals) and the 45S rDNA probe was detected using an avidin-FITC conjugate (Roche Biochemicals). All slides were counterstained with 2 μg mL^−1^ DAPI in Vectashield mounting medium (Vector Laboratories, Burlingame, CA).

For GISH experiments, *E. fulgens* and *E. puniceoluteum* genomic DNA was obtained following Ferreira and Grattapaglia (1998) and labeled, respectively, with biotin-14-dUTP and digoxigenin-11-dUTP by nick-translation (Roche Biochemicals). Initially, both probes were used simultaneously on *Epidendrum* hybrid metaphases, the *E. fulgens* probe was detected using avidina-FITC (Sigma) and the *E. puniceluteum* probe was detected using antidigoxigenin-Rodamina (Roche Biochemicals). A second experiment was performed using biotin-labeled *E. fulgens* DNA as a probe and unlabeled *E. puniceluteum* genomic DNA as a block at five different concentrations: 10×, 20×, 30×, 60×, and 90×, following Moraes and Guerra (2010).

#### Analysis and editing

All of the slides that were prepared using nonfluorescent stains (Giemsa and Alexander) were examined under an Olympus BX 50 microscope coupled with an Evolution™ MP camera (Media Cybernetics, Rockville, MD) and analyzed using the program Image ProPlus v6 (Media Cybernetics). Slides from CMA/DAPI banding, FISH and GISH, which used fluorescent stains, were examined using a DMRA2 epifluorescence microscope (Leica, London, UK), photographed with a Leica DCF365 FX camera and analyzed using the program LAS 3.0 (Leica). Pistil slides that were stained with aniline blue were examined with an Olympus BX 50 fluorescent microscope with a WU filter, photographed with an Olympus DP73 camera, and analyzed using the cellSens Entry software (Olympus). All images were uniformly processed for color balance, contrast, and brightness using Adobe Photoshop CS5 (Adobe Systems, San Jose, CA).

## Results

### Meiotic analysis

**Figure 2:**
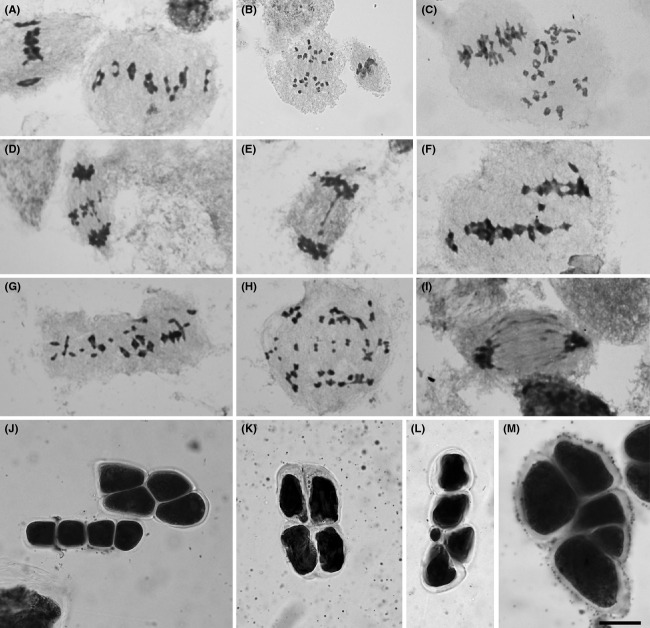
*Epidendrum* microsporogenesis. *Epidendrum fulgens* metaphase I (A) and anaphase I (B), *E. puniceoluteum* metaphase I and bivalents (C) and meiotic errors – chromosome lagging at anaphase I (D), anaphase I bridge (E), and metaphase I in two plains (F). Hybrid metaphase I showing 14 chromosome pairs and 10 univalents (G), anaphase I with lagging chromosomes at metaphase I (probably the same late univalent from G) and anaphase I with multiples bridges. Pollinium viability, estimated from tetrad stainability, showing a normal tetrad (J) and hybrid abnormalities, including wrinkling tetrad (K), micronuclei (L), and polyads (M). Scale bar in (M) indicates 10 μm.

The meiotic analysis of parental *E. fulgens* and *E. puniceoluteum* found high levels of meiotic normality – in average 99.60% and 96.06%, respectively – with chromosomes pairing as bivalents and following meiotic division forming four equal cells. The chromosome number could be determined in both species: *n* = 12 in *E. fulgens* (Fig. 2A and B) and *n* = 28 in *E. puniceoluteum* (Fig. 2C). A few abnormalities were observed in the parental slides, including unpaired chromosomes at prophase I/metaphase I and/or early disjunction at metaphase I in *E. fulgens* (0.37% and 0.31%, respectively) and unpaired chromosomes at prophase I/metaphase I and anaphase I bridge in *E. puniceoluteum* (0.58% and 0.48%, respectively; Fig. 2D and E; Table S1). An additional curious abnormality was observed in *E. puniceoluteum*: metaphase I with the metaphase plate divided in two (0.26%; Fig. 2F).

The meiotic analyses of hybrids (13 individuals from Cananéia and Ilha Comprida/SP) found lower levels of normality compared to the parental species (*H* = 24.019, *P* < 0.0001), with only 9.35%, in average, of cells carrying out normal meiosis. Nevertheless, two of 13 individuals had 88.8% and 87% normal meiotic cells, respectively, both presenting *n* = 19. The remaining 11 individuals had a mean normality of 0.12%. Unpaired chromosomes at metaphase I and anaphase I bridges were frequently observed; a consistent number of late chromosomes was observed, including 13–14 bivalents on the plate and 10–12 univalents; in anaphase I *c*. 13 chromosomes could be observed on poles and more than 12 chromosomes lagged on the metaphase plate (Fig. 2G–I; Table S1). However, 91.64% of the analyzed meiocytes presented complex abnormalities that were difficult to classify because they involved metaphase clumping/sticking of chromosomes with unpaired chromosomes.

#### Pollen viability and pollen tube growth

The pollen grains were delivered in tetrads inside a coherent mass, the massulae. Pollen grain stainability (strong/light purple stained) and morphology (normal/wrinkling) were examined to classify the grains as viable or unviable (Fig. 2L and M). The parental and hybrid individuals showed high percentages of normal strong stained pollen grains inside the tetrads, but hybrid viability (98.99%) was lower than observed in *E. fulgens* (99.78%; *H* = 15.1; *P* < 0.05; Table 1).

**Figure 3:**
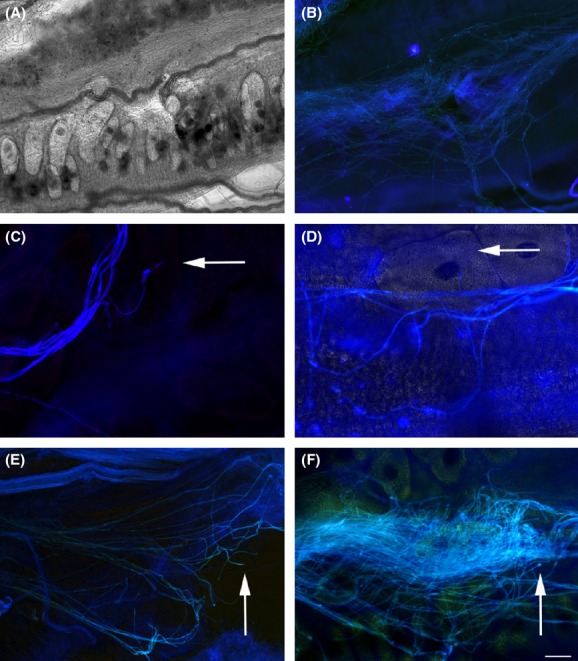
Pollen tube growth. (A and B) Pollen tube growth from crossings between *Epidendrum fulgens* × *E. fulgens* as a positive control – (A) light microscopy showing ovules and (B) fluorescent microscopy showing the pollen tube reaching the ovule level. (C and D) Pollen tube growth from crossings between *E. puniceoluteum* × hybrid (pollen receptor × donor) showing timid pollen tube germination reaching an ovule level. (E and F) Hybrid × *E. puniceoluteum* (pollen receptor × donor) showing pollen tubes reaching hybrid ovule level (arrows). Pollen tubes are in blue, acquired by fluorescence microscopy, and ovule photos were acquired by light contrast microscopy. (D–F) Merged images from fluorescence (pollen tube) and light microscopy (ovule). (A and C) Pollen tubes observed by fluorescence microscopy. Scale bar in (F) indicates 10 μm.

Pistils showed pollen tube growth only 12 days after hand-pollination in all crossings, except those involving *E. fulgens* and hybrid flowers, which were aborted soon after pollination and no flowers could be collected. Pollen tube growth was observed in all other crossings, with pollen tubes reaching to the ovules, but with differing intensities (Fig. 3). Positive control treatments showed intensive pollen tube growth, as was observed in *Epidendrum* hybrid (♀) × *E. puniceoluteum* (♂) crosses (Fig. 3A and B). However, when hybrids were used as pollen donors (*E. puniceoluteum* (♀) × *Epidendrum* hybrid (♂) and *Epidendrum* hybrid (♀) × *Epidendrum* hybrid (♂)), pollen tube germination was weak (Fig. 3C and F).

### Karyotype analysis – chromosome number, chromosome banding, and in situ hybridization

**Figure 4:**
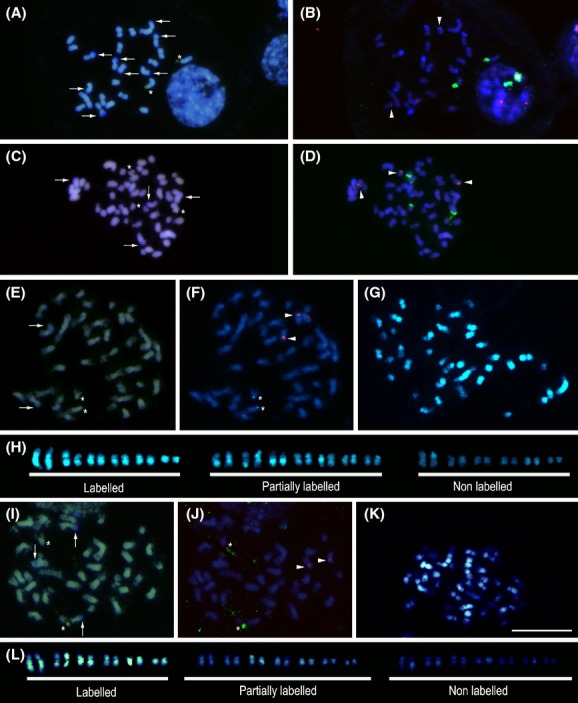
Chromosome banding (A, C, E, I), in situ hybridization (B, D, F, J), and GISH (G, H, K, L) for (A and B) *Epidendrum fulgens*, (C and D) *E. puniceoluteum*, (E–H) hybrid/2*n* = 38 and (I–L) hybrid/2*n* = 40. Arrows in (A, C, E, and I) indicate DAPI^+^ bands (blue) and asterisks indicate CMA^+^ bands (yellow). Arrowheads in (B, D, F, and J) indicate 5S rDNA (red) and asterisks show 45S rDNA (green). Scale bar in (K) is 10 μm.

In addition to counting the number of chromosomes through meiosis, it was also determined through mitosis, with 2*n* = 24 in *E. fulgens* and 2*n* = 56 in *E. puniceoluteum*. The hybrids chromosome numbers were defined here for the first time and presented an aneuploid variation of 2*n* = 38 and 2*n* = 40 (Fig. 4) (Table 2).

**Table 2:** *Epidendrum* karyotype

Species	Population	No. plants	2*n*	Chromosome banding		rDNA	
	
				CMA^+^	DAPI^+^	5S	45S
*E. fulgens*	Paraty/RJ	2	24	2 ter, met	8–10, ter met	2 prox, sbmet	2 ter, met
	Florianópolis/SC	1					
*E. puniceoluteum*	Ilha Comprida/SP	2	56	2 ter + 2 int	2 ter + 2 subter, met	3 prox, sbmet	3 ter, met
	Paranaguá/PR	2					
	Cananéia/SP	1					
*E. hybrid*	Imbituba/SC	1	40	2 ter, met	2 ter + 1 subter, met	2 prox, sbmet	2 ter, met
	Ilha Comprida/SP	3					
	Cananéia/SP	3					
	Ilha Comprida/SP	4	38	2 ter, met	2 ter, met		
	Cananéia/SP	4					

Chromosome morphology: met, metacentric; sbmet, submetacentric. Signal position: ter, terminal; subter, subterminal; int, interstitial; prox, proximal.

#### CMA/DAPI banding

The CMA/DAPI banding revealed a diverse band pattern, with DAPI^+^ band variability (Table 2). *Epidendrum fulgens* had eight (sometimes up to 10) terminal DAPI^+^ bands and two CMA^+^ bands (Fig. 4A), while *E. puniceoluteum* had four DAPI^+^ bands (two terminals and two subterminals) and three CMA^+^ band (Fig. 4C). Among the hybrids, two (one terminal and one subterminal) and three (one terminal and two subterminal) DAPI^+^ bands were observed in individuals with 2*n* = 38 and 2*n* = 40, respectively (Fig. 4E and I). The CMA^+^ band pattern was more conservative, with two terminal bands in *E. fulgens* and hybrids, and three terminal bands in *E. puniceoluteum*, in addition to tiny proximal bands on all chromosomes and hybrids (Fig. 4A, C, E, and I).

#### 5S and 45S rDNA

Two proximal 5S rDNA and two terminal 45S rDNA sites were observed in *E. fulgens* (Fig. 4B), while in *E. puniceoluteum*, three proximal 5S rDNA and three terminal 45S rDNA sites were observed (Fig. 4D). In both species, 45S rDNA sites were colocalized with terminal CMA^+^ bands. Among the hybrids, all individuals (with 2*n* = 38 and 2*n* = 40) had two proximal 5S rDNA and two terminal 45S rDNA sites that were colocalized with CMA^+^ terminal bands (Fig. 4F and J).

### GISH

GISH on hybrid mitotic metaphases using *E. fulgens-* and *E. puniceoluteum-*labeled genomic DNA as probes, without blocking DNA, failed to differentiate the two subgenomes; all of the chromosomes were labeled uniformly (data not shown). However, when *E. fulgens-*labeled DNA was used as a probe and *E. puniceoluteum*-unlabeled genomic DNA was used for blocking (1:30), differential labeling was observed on hybrid metaphases. In 2*n* = 38 individuals, 12 completely labeled chromosomes, 14 partially labeled chromosome, and 12 unlabeled chromosomes were observed (Fig. 4G and H). However, in 2*n* = 40 individuals, 12 completely labeled chromosomes, 14 partially labeled chromosome, and 14 unlabeled chromosomes were observed (Fig. 4K and L).

## Discussion

The study of hybrid zones between flowering plant species have provided important results regarding the evolution of reproductive barriers (Widmer et al. 2009), the role of habitat selection in diverging lineages (Johnston et al. 2001; Marques et al. 2010), and the process of speciation (Abbott et al. 2013). As differences in chromosome numbers and ploidy levels are historically considered strong isolating barriers among species (Stebbins 1971; Coyne and Orr 2004), few studies have investigated putative hybrid zones between species showing such differences. However, recent studies have provided data showing extensive hybrid zones presenting high levels of interploidal gene exchange (Ramsey and Schemske 1998; Petit et al. 1999; Chapman and Abbott 2010). The results of this study support the findings of Pinheiro et al. (2010), which characterized an extensive hybrid zones between two species with different ploidy levels: *E. fulgens* (2*n* = 2*x* = 24) and *E. puniceoluteum* (2*n* = 4*x* = 56). Here, we observed that despite an almost completely abnormal meiosis and lower pollen viability, hybrids still present few pollen tube growths when used as pollen donors in controlled crossing experiments. However, the introgression was unidirectional toward just one parental, *E. puniceoluteum*.

The karyotype analyses indicate a reduction on amount of repetitive DNA (heterochromatic blocks and rDNA sites) on hybrid genomes, in agreement to expect diploidization event. Chromosome doubling is assumed to be one of the first events during the diploidization process, ensuring the fertility restoration (Soltis et al. 2003, 2010; Soltis and Soltis 2009), but until now it has not occurred in these hybrids, and the hybrid are still triploids. These unexpected finding highlight the importance of studies investigating hybrid zones formed by species with different ploidy, challenging the widely held view of ‘instant isolation’ among species of different ploidy (Coyne and Orr 2004).

The chromosome number observed in *E. fulgens* (*n* = 12/2*n* = 2*x* = 24) agreed with previous reports (Blumenschein 1960; Tanaka and Kamemoto 1984, Pinheiro et al. 2009), but the chromosome number observed in *E. puniceoluteum* (*n* = 28/2*n* = 4*x* = 56) were not the same of earlier reports of 2*n* = 52 (Pinheiro et al. 2009). The most likely explanation for variation in chromosome number of *E. puniceoluteum* is the occurrence of two cytotypes. This possibility has direct consequences for the hybrid swarm, especially considering the observation of two chromosome numbers among hybrids: 2*n* = 38 (supposedly 12 *E. fulgens* chromosomes and 26 *E. puniceoluteum* chromosomes) and 2*n* = 40 (supposedly 12 *E. fulgens* chromosomes and 28 *E. puniceoluteum* chromosomes). In fact, the variation of the chromosome number in hybrids and GISH results suggest the additional chromosome pair in 2*n* = 40 individuals came from the *E. puniceoluteum* because the 12 well labeled chromosomes that came from *E. fulgens* were constant between the 2*n* = 38 and 40 individuals.

Another possibility for this aneuploid variation is meiotic errors in *E. puniceoluteum*, despite the high meiotic normality index (97%; Table 1). A closely related polyploid species, *E. cinnabarinum* Salzm., had a reduced meiotic normality index (66% in meiosis II) and low tetrad viability (8.2%), suggesting an unbalanced gamete formation (Da Conceição et al. 2006). However, considering that no aneuploid gametes were observed in *E. puniceoluteum* and that crossings involving hybrids as the pollen donor did not form seeds (Pinheiro et al. 2010), hybrid aneuploid variation could be a combined consequence of the effect of hybrid meiosis abnormalities on ovule formation and *E. puniceoluteum* backcrossing.

If interploidal hybrids were completely sterile, they could represent a genetic dead end of little evolutionary relevance (Mayr 1942), but even with low fitness, hybrids may act as a conduit for genetic exchange and could have substantial impacts on the population (Husband et al. 2008; Arnold et al. 2012). The 98.99% of viable tetrads in hybrids is probably an overestimation, consequence of staining technique employment to estimate pollen viability, but the data from hybrid pollen tube germination is a stronger indicative of low hybrid pollen viability and the strong postpollination barrier between hybrid and *E. fulgens*, but not so strong between hybrid and *E. puniceoluteum*. In fact, previous studies by Pinheiro et al. (2010) have demonstrated asymmetric introgression toward *E. puniceoluteum*, but always with this species as the pollen donor.

Hybrid fertility is expected to be a function of the percentage of meiotic paired chromosomes –the higher the number of univalents and multivalents, the lower the fertility (Levin 2002) – plus allelic interactions among fertility genes (Buerkle et al. 2000; Bomblies et al. 2007; Chase 2007; Rieseberg and Blackman 2010; He et al. 2011). No multivalents were observed in the hybrids examined here, but univalents were currently detected (*c*. 10–12). Despite the fact that the hybrid is triploid and the presence of univalents, some chromosome pairing was detected, ensuring some gamete production, probably unbalanced ones, with a portion of the univalents going together to one side or the other during anaphase I or even forming a separate gamete. As such triploid gamete production is expected to be very low, we can consider the possibility that *E. puniceoluteum* pollen grains (*n* = 28) have fertilized different unbalanced hybrid gametes (*n* = 10 and *n* = 12), which gave rise to new hybrid backcrosses with 2*n* = 38 and 2*n* = 40. Similar patterns were suggested from analyses of triploids formed by *Dactylohriza fuchsia* (2*n*) × *D. praetermissa* (4*n*) and *D. fuchsia* (2*n*) × *D. purpurella* (4*n*), with the conclusion being that a consistent number of 20 univalents occurred during hybrid metaphase I, which would tend to be grouped in an aneuploid gamete (Heslop-Harrison 1953). However, Lord and Richards (1977) examined hybrids formed by *D. fuchsia* (2*n*) × *D. purpurella* (4*n*) and found a variable univalent number, between 9 and 12, and 38% aneuploid hybrids.

It is accepted that backcrossing occurs more often between a triploid hybrid and its diploid parental (Ramsey and Schemske 1998), and that such interspecific crosses are more fertile when the female gamete contains more chromosomes than the male gamete (Stebbins 1971; Soltis et al. 2003; Slotte et al. 2008; Erilova et al. 2009; Jorgensen et al. 2011). This is thought to be related to the maternal:paternal (2:1) genome balance in the endosperm, which plays an important role in hybrid viability (Johnston et al. 1980; Erilova et al. 2009). However, because Orchidaceae has no endosperm, this could facilitate crossing in both directions. As observed here, a triploid hybrid that formed from diploid *D. incarnata* and tetraploid *D. lapponica* could only backcross with the tetraploid parental (but authors did not indicate the direction of crossing; Aagaard et al. 2005), contradicting the previous triploid x diploid preferential crossing (Ramsey and Schemske 1998). Also introgression between *D. fuchsii* (2*n*) and *D. maculata* (4*n*) was observed in both directions (Ståhlberg 2009). Moreover, the pollen grain conformation as a cohesive mass of tens of tetrads, the pollinium, can have a direct influence on hybrid reproductive success (Harder and Johnson 2008) by minimizing the loss of gametes. Because a pollinium is deposited on a stigma as an entire unit, it reduces pollen loss, and the highly abnormal hybrid meiosis (which should produce only a few viable female gametes) could be compensated for by a massive *E. puniceoluteum* pollen deposition, which should pollinate any viable female hybrid gametes.

Three hybrid individuals (two with 2*n* = 40 and one with 2*n* = 38) of the 23 analyzed here were initially identified by nuclear SSR loci as *E. puniceoluteum* individuals (Pinheiro et al. 2010), indicating that a high incorporation of parental genome sequences can occur into late hybrids as a consequence of recurrent backcrossing. The same pattern was observed in a sympatric population of *Serapias* (Orchidaceae), which was analyzed by amplified fragment length polymorphism (AFLP), in which many plants classified as parental species were actually introgressed hybrids, suggesting that the co-occurring *Serapias vomeracea* and *S. cordigera* undergo extensive interspecific gene flow and hybrid backcrossing (Bellusci et al. 2010). Such recurrent long-term hybridization and introgression events could contribute to increasing biodiversity, as observed in bromeliads species (Palma-Silva et al. 2011).

**Figure 5:**
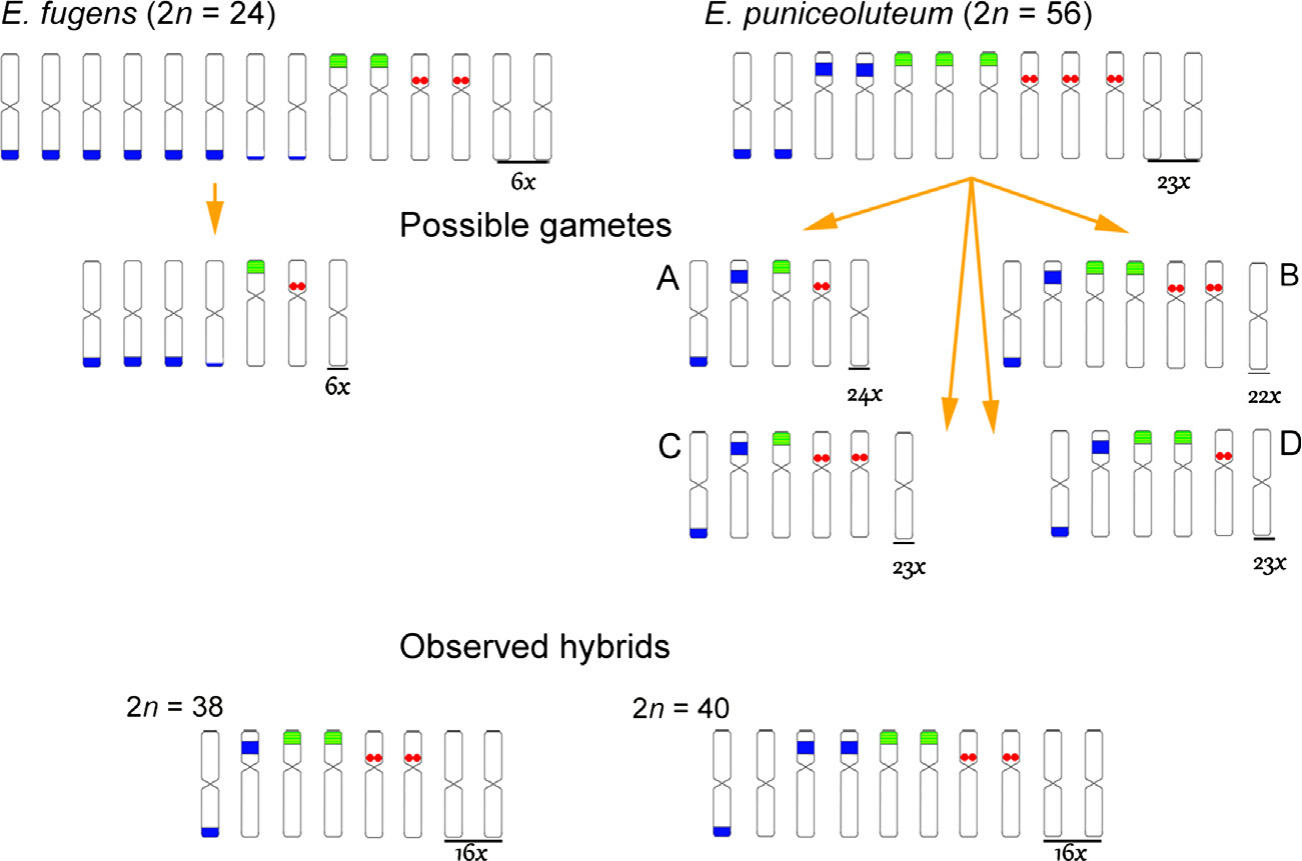
Karyotype of *Epidendrum fulgens*, *E. puniceoluteum* and hybrid; and possible gametes formed by *E. fulgens* and *E. puniceoluteum* to form hybrids. Blue blocks indicate DAPI^+^ bands; green blocks show CMA^+^ bands/45S rDNA sites; red blocks indicate 5S rDNA sites. Number below chromosomes on gametes and chromosome pairs on karyotypes indicate number of repetition of such unlabeled chromosomes. Letters A–D indicate four possible gametes formed by *E. puniceoluteum*.

All hybrids presented 2*n* = 38 or 40, showing a nonrandom distribution of chromosome number and suggesting that certain somatic chromosome combinations are more viable than others, possibly reflecting nonrandom hybrid meiotic products (Lord and Richards 1977). In the hybrids, the DAPI^+^ bands were reduced compared to the expected number of bands, with just two (2*n* = 38) and three (2*n* = 40) bands being observed (for total parental DAPI^+^ bands and possible gametes see Fig. 5). Two sites for each of 5S and 45S rDNA were observed in all hybrids, but considering the *E. puniceoluteum* karyotype, one could even expect three sites (for expected patterns of 5S and 45S rDNA, see Fig. 5). Hybridization events are usually followed by a diploidization process, in which repetitive DNA suffers a dynamic reorganization by expansion/reduction of sequences, which could explain the reduction in the repetitive DNA sequences (heterochromatic blocks and rDNA sites) in hybrids (Clarkson et al. 2005; Leitch and Leitch 2008; Renny-Byfield et al. 2013), in addition to alterations in the DNA methylation pattern (Paun et al. 2009, 2010; Flatscher et al. 2012). The time required to complete the diploidization process can be determined from patterns of epigenetic variation, as observed in *Dactylohriza* (Paun et al. 2011b), but also GISH patterns (Lim et al. 2004; Renny-Byfield et al. 2013). The hybrid formation examined here is not thought to be a recent event (Pinheiro et al. 2010), but not old enough to GISH fail and hybrid/parental complete isolation (Clarkson et al. 2005; Koukalova et al. 2010). Estimating time in generations, complete hybrid/parental separation requires *c*. 60 generations (Rieseberg and Willis 2007).

Note that the hybrid individuals examined here have not duplicated their chromosomes. However, hybrid speciation that starts from interploidal crossing is particularly prone to resulting in allopolyploids (Chapman and Burke 2007; Buggs et al. 2009; Paun et al. 2009, 2011b). Some hybrids are formed between closely related species with the same ploidy level and represent introgressed ecological forms that colonize diverse habitats and lack postzygotic isolation from their parents, as in the hybrid examined here. However, the hybrid presented here is a triploid formed by interploidal crossing, but curiously it is able to backcross with one parental.

Even without duplicating their chromosomes and having low fertility, the *Epidendrum* hybrids largely colonize both parental habitats (the sand dunes of *E. fulgens* and the swampy regions of *E. puniceoluteum*, both along the *restinga* vegetation on the Brasilian seashore; Pinheiro et al. 2010), suggesting that hybridization might increase ecological flexibility or colonization ability (Petit et al. 1999). In the present hybrids, their vegetative reproduction and perennial habits should have contributed to their persistence in the habitats of both parental species (Pinheiro et al. 2010).

The hybrid GISH pattern was able to separate the chromosomes into three groups: well labeled (originated from *E. fulgens*), poorly labeled, and nonlabeled (both originated from *E. puniceoluteum*). The two latter groups indicated that *E. puniceoluteum* chromosomes share some *E. fulgens* sequences (poorly labeled chromosomes). During the diploidization period, retrotransposons and DNA satellites jump from one genome to another and begin hybrid genome homogenization, what could explain these 14 constant *E. puniceoluteum* chromosomes that were poorly labeled by the *E. fulgens* genome probe. An alternative explanation could be a hybrid origin for *E. puniceoluteum*, with *E. fulgens* as one of its parents. Throughout its distribution, *E. fulgens* overlap with other species (i.e., sympatric zones with *E. denticulatum* and *E. secundum* that contain putative hybrids; Pinheiro et al. 2009), and *E. puniceoluteum* could have originated from one of these *E. fulgens* sympatric zones.

The pattern of introgression between *E. fulgens* and *E. puniceoluteum* challenges the widely held view of hybrid ‘instant isolation’ and the polyploidy tendency after divergent parental crossing (Coyne and Orr 2004; Chapman and Burke 2007; Buggs et al. 2009; Paun et al. 2011b), and it opens interesting research possibilities throughout the *Epidendrum* sympatric zones for investigating the evolutionary potential of interploidal hybridization. In addition, both species and their hybrids are distributed throughout the *restinga* vegetation, an harsh environment in which plants face flooding, drought, constant wind, high salinity, a lack of nutrients, and a broad ecological amplitude (Scarano 2002). Considering these conditions, the adaptive component of these extensive hybridization zones is of particular note. Next-generation sequencing techniques could improve the detection of specific genomic regions that will be useful for improving our understanding of genomic reorganization after hybridization (Chester et al. 2010, 2012; Buggs et al. 2012), especially after this intriguing interploidal crossing resulted in diploid hybrids, and for localizing sequences associated with selection for divergent habitats, which also occurs in hybrid genomes (Twyford and Ennos 2012) and may be associated with the broad ecological amplitude and high frequency of hybrids observed in natural populations.

## Supporting Information

**Figure S1.** *Epidendrum fulgens* – organism photograph

**Table S1.** Meiotic abnormalities in Epidendrum. Number and proportion (%) of each meiotic abnormality is shown for *E. fulgens, E. puniceoluteum* and hybrids.
