# Supplementary figures and images for: Interploidy hybridization in sympatric zones: the formation of Epidendrum fulgens × E. puniceoluteum hybrids (Epidendroideae, Orchidaceae)
Source: Ecol Evol. 2013 Sep 12;3(11):3824–37. doi: 10.1002/ece3.752 (PMC3810877; doi:10.1002/ece3.752)

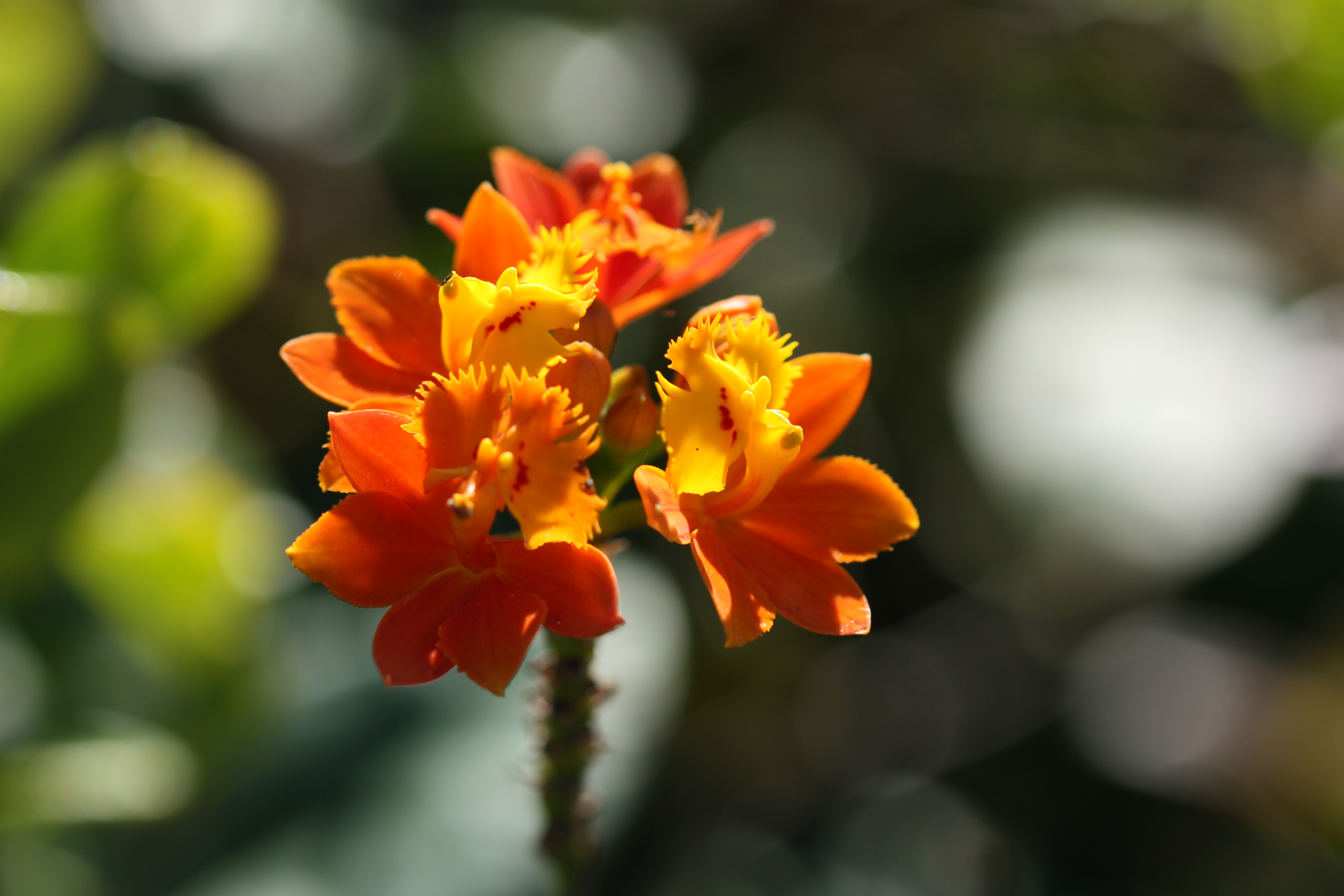

Supplement: Supplementary file 1 [file ece30003-3824-SD1.jpg]
