# Supplementary material for: Interploidy hybridization in sympatric zones: the formation of Epidendrum fulgens × E. puniceoluteum hybrids (Epidendroideae, Orchidaceae)
Source: Ecol Evol. 2013 Sep 12;3(11):3824–37. doi: 10.1002/ece3.752 (PMC3810877; doi:10.1002/ece3.752)
Supplement: Supplementary file 2 [file ece30003-3824-SD2.doc]

| Species | N. cell | Meiotic Abnormalities (%) | | | | | | | | | | | | | |
| --- | --- | --- | --- | --- | --- | --- | --- | --- | --- | --- | --- | --- | --- | --- | --- |
|  |  | Univalent / Tetravalents | Unpaired chromosomes at metaphase I | Metaphase I divided in two plans | Bivalent early disjuction | Unequal segregation | Prophase II with micronuclei | Prophase II with bridge | Anaphase I lagging chromosomes | Anaphase I bridge | dyads | Tryads | Tetrad with Micronuclei / microcyte | Polyad (Five nuclei) | Complex abnormalities* |
| *Epidendrum fugens* | 3,000 | 2 (0.06) | 13 (0.37) | - | 11 (0.31) | - | - | - | 1 (0.03) | - | - | 2 (0.06) | - | - | - |
| *Epidendrum puniceluteum* | 4,000 | 1 (0.02) | 24 (0.48) | 13 (0.26) | 3 (0.06) | - | - | - | 18 (0.36) | 29 (0.58) | - | 2 (0.04) | 23 (0.46) | - | - |
| Hybrid | 7,500 | 9 (0.12) | - | - | - | 2 (0.03) | 18 (0.24) | 2 (0.03) | - | 73 (0.97) | 15 (0.2) | 17 (0.23) | 14 (0.19) | 2 (0.03) | 6441 (85.88) |

*Chromosome clumping/sticking with unpaired chromosomes at Metaphase I/Anaphase I
